# Fecal calprotectin predicts complete mucosal healing and better correlates with the ulcerative colitis endoscopic index of severity than with the Mayo endoscopic subscore in patients with ulcerative colitis

**DOI:** 10.1186/s12876-017-0669-7

**Published:** 2017-10-23

**Authors:** Sun-Ho Lee, Min-Ju Kim, Kiju Chang, Eun Mi Song, Sung Wook Hwang, Sang Hyoung Park, Dong-Hoon Yang, Kyung-Jo Kim, Jeong-Sik Byeon, Seung-Jae Myung, Suk-Kyun Yang, Byong Duk Ye

**Affiliations:** 1 Department of Gastroenterology, University of Ulsan College of Medicine, Asan Medical Center, 88, Olympic-ro 43-gil, Songpa-gu, Seoul, 05505 South Korea; 2 Department of Clinical Epidemiology and Biostatistics, University of Ulsan College of Medicine, Asan Medical Center, 88, Olympic-ro 43-gil, Songpa-gu, Seoul, 05505 South Korea; 3 Inflammatory Bowel Disease Center, University of Ulsan College of Medicine, Asan Medical Center, 88, Olympic-ro 43-gil, Songpa-gu, Seoul, 05505 South Korea

**Keywords:** Calprotectin, Ulcerative colitis, Endoscopic score

## Abstract

**Background:**

We aimed to evaluate the role of fecal calprotectin (FC) as a noninvasive marker for the disease activity of ulcerative colitis (UC) in a Korean cohort.

**Methods:**

A total of 181 fecal samples were collected from 181 consecutive UC patients between April 2015 and September 2016. FC levels were measured using the Quantum Blue^®^ Calprotectin rapid test. The laboratory test results, partial Mayo Score (pMS), and colonoscopic imaging findings at FC level measurement were retrospectively reviewed. The Mayo endoscopic subscore (MES) and UC endoscopic index of severity (UCEIS) were graded by 2 certified endoscopists after training with 50 other cases.

**Results:**

The FC levels were significantly correlated with pMS (Spearman correlation coefficient *r* = 0.428, *p* < 0.001), MES (*r* = 0.304, *p* < 0.001), UCEIS (*r* = 0.430, *p* < 0.001), and CRP (*r* = 0.379, *p* < 0.001). FC levels exhibited a significantly better correlation with UCEIS than with MES (Meng’s z = − 2.457, *p* = 0.01). The FC cut-off level of 187.0 mg/kg indicated complete mucosal healing (MES = 0; UCEIS =0) with a sensitivity and specificity of 0.857 and 0.891, respectively (area under the curve, 0.883; 95% confidence interval, 0.772–1.000).

**Conclusion:**

The FC level is significantly correlated with the clinical disease activity index, endoscopic indices, and serum inflammatory biomarkers in a Korean UC cohort. FC is highly predictive of complete mucosal healing in UC. UCEIS exhibits a stronger correlation with the FC level, as compared to MES. Thus, FC could be used as a reliable noninvasive indicator for evaluating disease activity and mucosal healing in UC.

## Background

Ulcerative colitis (UC) is a chronic inflammatory bowel disease (IBD) characterized by a disease course involving relapses and remissions [1]. Historically, clinical remission was the major treatment target for patients with UC. However, due to the unreliability and inaccuracy of symptoms indicating actual mucosal inflammation, there has been a paradigm shift towards therapeutically targeting more objective parameters such as mucosal healing (MH) in addition to other clinical endpoints [2–7]. In fact, MH is reportedly more strongly associated with both short-term and long-term outcomes in patients with UC [4, 7–9].

However, the use of repeated endoscopy to verify MH would be invasive, inconvenient, and expensive, and may present a risk of significant complications (i.e. colonic perforation). Therefore, noninvasive surrogate markers indicating endoscopic healing have been investigated to replace the repeated endoscopic procedures. Among these surrogate markers, fecal calprotectin (FC) has reportedly shown significant correlations with both clinical and endoscopic activity indices in patients with UC in several recent studies [10–14].

Among the endoscopic activity indices of UC, the Mayo endoscopic subscore (MES)—although not validated—is the most widely used index [15]. In addition, the ulcerative colitis endoscopic index of severity (UCEIS) has been recently developed and validated as a reliable endoscopic activity index in patients with UC [16, 17]. Although the correlation of FC levels with both MES and UCEIS has been previously evaluated by Theede et al. [14], a direct comparison between these correlations has not been described thus far. Moreover, only a few studies in Asia have evaluated the use of FC as a surrogate marker for disease activity in UC patients [18–23], and most of these reports were limited by their small sample size.

In the present study, we aimed to evaluate the diagnostic role of FC as a noninvasive marker for the disease activity of UC and to compare the correlations of FC with MES and UCEIS in a large Korean cohort.

## Methods

### Study population

Among patients with UC managed at Asan Medical Center (a tertiary care center in Seoul, Korea), a total of 181 consecutive patients who underwent FC measurements between April 2015 and September 2016 were enrolled in this study; all of these patients were of Korean descent. UC was diagnosed based on the current standard clinical, radiological, endoscopic, and histopathological criteria [24, 25]. A total of 181 fecal samples were collected from 181 UC patients. If repeated fecal samples were collected from a single patient, the first FC level measurement was used for the study. Detailed demographic and clinical information were retrieved from the electronic medical records and from the Asan IBD registry, which has been prospectively maintained since 1997 and has been previously described in detail [26, 27]. Collected data included birth date, sex, date of UC diagnosis, family history of IBD, smoking status, maximum disease extent, and medications. At our institute, all UC patients are instructed to record their bowel frequency and degree of rectal bleeding for 3 days before visiting the clinic to facilitate the measurement of the partial Mayo Score (pMS) [15]. During their visit, the pMS was calculated based on the patients’ records and physician’s global assessment using a computerized program incorporated into our electronic medical record system, and the value was recorded in the clinical note. When evaluating the correlation between FC levels and pMS, only the pMS values within 6 weeks of the FC measurement were used. The median time interval between FC and pMS evaluation was 2 days (interquartile range [IQR], 1-7 days).

### Laboratory and endoscopic evaluation

The following laboratory parameters were recorded at the time of FC level measurement: complete blood cell count (XE-2100™, Sysmex, Kobe, Japan), including white blood cell count (normal range [NR], 4–10 × 10^3^/μL), hemoglobin level (NR, 12–16 g/dL), hematocrit value (NR, 36–48%), and platelet count (NR, 150–350 × 10^3^/μL); erythrocyte sedimentation rate (ESR; NR for men: 0–9 mm/h; NR for women: 0–20 mm/h; TEST 1, Alifax, Padova, Italy); and serum chemistry values (Cobas 8000 modular analyzer, Roche Diagnostics, Basel, Switzerland; AU5800 Beckman Coulter, Brea, CA), including C-reactive protein (CRP; NR, 0–0.6 mg/dL) and albumin (NR, 3.5–5.2 g/dL) levels. The time interval between the aforementioned laboratory tests and FC level measurement was within 2 months (median time interval, 0 day; IQR, 0-1 day).

The endoscopic images (full colonoscopic [75 cases] or flexible sigmoidoscopic [106 cases] images) obtained at the time of FC level measurement were reviewed by 2 board-certified endoscopic experts (B.D.Y. and S.W.H.) who were blinded to the clinical details, including FC levels and laboratory results. The time interval between the endoscopic procedure and FC level measurement was within 4 months (median time interval, 2 days; IQR, 0-15 days). All the endoscopic images were stored in the picture archiving and communication system (PACS) of Asan Medical Center. The 2 reviewers independently evaluated the endoscopic images and determined the MES and UCEIS [15, 16]. In case of a disagreement regarding the MES and UCEIS values between the 2 reviewers, a final score was recorded based on a consensus between the 2 reviewers. Prior to reviewing the 181 study cases, the endoscopic experts were trained using training set images of colonoscopy or flexible sigmoidoscopy from 50 other cases; during this training exercise, the reviewers independently determined the MES and UCEIS for 50 other cases, and consensus was sought regarding the scoring between the 2 reviewers to enhance the agreement in their scoring.

### Analysis of the fecal calprotectin level

Patients were instructed to collect their fecal samples. Fecal samples were collected at the clinic or at home and were stored in a refrigerator (2–8 °C) when collected at home. The samples were sent to the department of laboratory medicine at Asan Medical Center within 1 day of collection for analysis. The laboratory personnel were blinded to the clinical data, laboratory values, and endoscopic findings of the patients. FC levels were measured using the Quantum Blue^®^ Calprotectin rapid test (Bühlmann Laboratories AG, Schönenbuch, Switzerland), and high-range kit (measuring 100–1800 mg/kg) was used. All samples with FC levels exceeding the assay range of 1800 mg/kg were reanalyzed after dilution of the samples according to the manufacturer’s instructions for the exact measurement of the FC values. Cases with FC levels below the assay range (<100 mg/kg) were set as 100 mg/kg.

### Statistical analysis

The continuous variables were reported as medians and interquartile ranges (IQR), whereas the categorical variables were reported as numbers and percentages. The inter-rater agreement of the endoscopic indices was analyzed using Cohen’s weighted Kappa coefficient. Correlation analysis was performed using Spearman’s rank correlation coefficient. The comparison between the coefficients of correlations of MES and UCEIS with the FC levels was conducted using Meng’s z coefficient [28]. The receiver operating characteristic (ROC) curves for the FC levels were assessed to predict the clinical and endoscopic activity indices. The comparison between the area under the ROC curve was analyzed using DeLong’s test [29]. A *p* value of <0.05 was considered statistically significant. All statistical analyses were performed using SPSS 21.0 for Windows (IBM SPSS Statistics, Ver. 21.0; IBM Co., Armonk, NY) and R V.3.4.0 (R Development Core Team).

## Results

### Patient characteristics

Among 181 study subjects, 107 (59.1%) were men, and the median age at diagnosis of UC was 35.8 years (IQR, 26.1–47.8 years; Table 1). The median age at FC level measurement was 40.5 years (IQR, 29.2–53.8 years) and the median disease duration prior to FC level measurement was 18.4 months (IQR, 1.9–59.5 months). The proportion of proctitis, left-sided colitis, and extensive colitis in the cohort was 18.2%, 26.0%, and 55.8%, respectively. Regarding severity of UC, 116 patients (64.1%) had moderate or severe disease activity. With regard to the history of UC medication use, 154 (85.1%), 116 (64.1%), and 124 (68.5%) patients were administered oral 5-aminosalicylic acids, topical 5-aminosalicylic acids, and systemic corticosteroids, respectively (Table 1). Fifty-eight (32.0%) and 30 (16.6%) patients were treated with thiopurines and anti-tumor necrosis factor-α agents, respectively (Table 1).

**Table 1:** Demographic and clinical characteristics of the study subjects

Variables	UC (*N* = 181)
Sex	
Male	107 (59.1%)
Age at UC diagnosis (years), median (IQR)	35.8 (26.1–47.8)
Age at FC level measurement (years), median (IQR)	40.5 (29.2–53.8)
Disease duration before FC level measurement (months), median (IQR)	18.4 (1.9–59.5)
Maximum extent of UC	
Proctitis	33 (18.2%)
Left-sided colitis	47 (26.0%)
Extensive colitis	101 (55.8%)
Disease severity of UC	
Clinical remission (Mayo score 0-2)	17 (9.4%)
Mild activity (Mayo score 3-5)	48 (26.5%)
Moderate activity (Mayo score 6-10)	98 (54.1%)
Severe activity (Mayo score 11-12)	18 (10.0%)
Smoking status at UC diagnosis	
Never smoker	100 (55.2%)
Former smoker	47 (26.0%)
Current smoker	34 (18.8%)
Family history of IBD	
No family history	171 (94.5%)
First-degree relative	9 (5.0%)
Second-degree relative	1 (0.5%)
History of medication use at FC level measurement	
Oral 5-ASA	154 (85.1%)
Topical 5-ASA	116 (64.1%)
Topical steroids	32 (17.7%)
Systemic corticosteroids	124 (68.5%)
Thiopurines	58 (32.0%)
Cyclosporine	4 (2.2%)
Anti-TNF agents	30 (16.6%)
FC level (mg/kg), median (IQR)	1518.0 (360.0–4205.0)
Hematocrit value (%), median (IQR)	37.7 (32.8–42.0)
Serum albumin level (g/dL), median (IQR)	3.7 (2.9–4.0)
Erythrocyte sedimentation rate (mm/h), median (IQR)	26.0 (11.5–44.5)
White blood cell count (×10^3^ /uL), median (IQR)	7.1 (5.6–9.5)
Platelet count (×10^3^ /uL), median (IQR)	300.0 (242.0–379.0)
C-reactive protein level (mg/L), median (IQR)	0.41 (0.10–2.37)
Partial Mayo score, median (IQR)	5 (2–6.5)
Mayo endoscopic subscore, median (IQR)	3 (2–3)
Mayo score, median (IQR)	6 (3–8)
UCEIS, median (IQR)	4 (3–6)

*UC* ulcerative colitis, *FC* fecal calprotectin, *IBD* inflammatory bowel disease, *TNF* tumor necrosis factor, *N* Number, *IQR* interquartile range, *ASA* aminosalicylic acid, *UCEIS* ulcerative colitis endoscopic index of severity

The median FC level was 1518.0 mg/kg (IQR, 360.0–4205.0 mg/kg), whereas the median pMS, MES, and UCEIS values were 5 (IQR, 2–6.5), 3 (IQR, 2–3), and 4 (IQR, 3–6), respectively (Table 1).

### Correlation of the fecal calprotectin level with biochemical markers

**Fig. 1:**
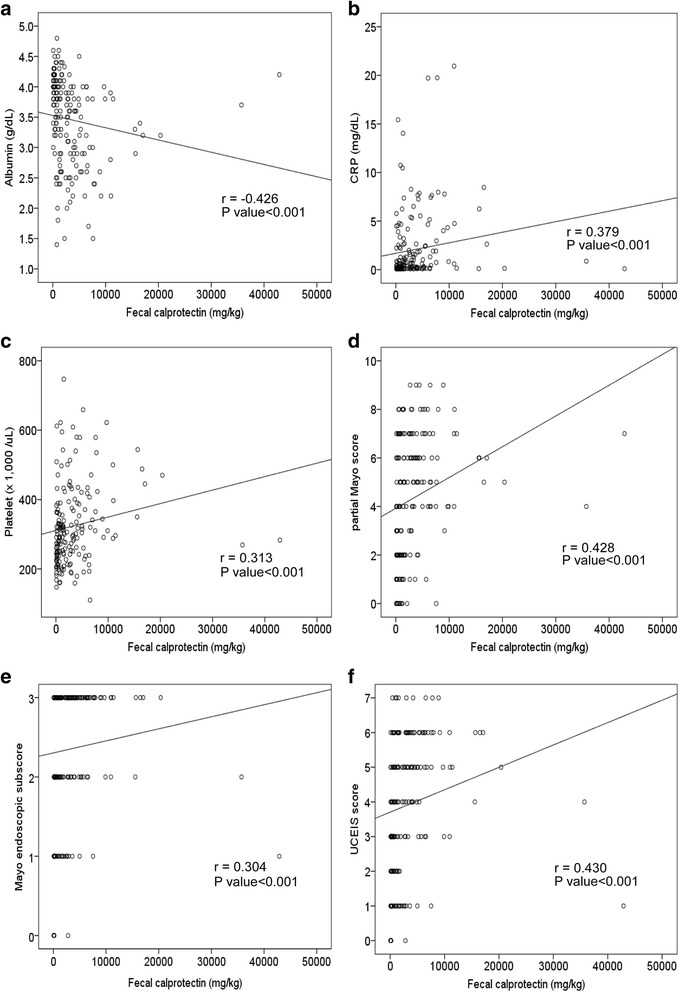
Correlation between fecal calprotectin levels and **a**) serum albumin levels (*r* = −0.426, *p* < 0.001); **b**) CRP levels (*r* = 0.379, *p* < 0.001); **c**) platelet counts (*r* = 0.313, *p* < 0.001); **d**) partial Mayo Score (*r* = 0.428, *p* < 0.001); **e**) Mayo endoscopic subscore (*r* = 0.304, *p* < 0.001); and **f**) UCEIS (*r* = 0.430, *p* < 0.001). *r* Spearman’s correlation coefficient, *CRP* C-reactive protein, *UCEIS* ulcerative colitis endoscopic index of severity

Among the biochemical markers, FC levels exhibited significant correlations with the CRP (*r* = 0.379, *p* < 0.001) and serum albumin (*r* = − 0.426, *p* < 0.001) levels (Fig. 1). The other laboratory values including white blood cell count (*r* = 0.231, *p* = 0.001), hematocrit value (*r* = − 0.238, *p* = 0.001), platelet count (*r* = 0.313, *p* < 0.001), and ESR (*r* = 0.249, *p* < 0.001) exhibited weaker correlations with the FC levels, as compared to both the CRP and serum albumin levels (Fig. 1).

### Correlation of the fecal calprotectin level with the disease activity index and endoscopic indices

The Cohen’s weighted Kappa coefficient of inter-rater agreement for MES and UCEIS were 0.78 (95% confidence interval [CI], 0.71–0.85) and 0.62 (95% CI, 0.56–0.69), respectively (Table 2). The Cohen’s weighted Kappa coefficient of the ‘vascular’, ‘bleeding’, and ‘erosions and ulcers’ items of the UCEIS were 0.69 (95% CI, 0.56–0.69), 0.40 (95% CI, 0.31–0.49), and 0.74 (95% CI, 0.67–0.81), respectively. The UCEIS and MES showed a strong correlation with each other (*r* = 0.876, *p* < 0.001). The FC levels exhibited significant correlations with the pMS (Spearman correlation coefficient *r* = 0.428, *p* < 0.001), MES (*r* = 0.304, *p* < 0.001), and UCEIS (*r* = 0.430, *p* < 0.001) (Fig. 1). When comparing the degree of correlation between the FC levels and endoscopic indices, UCEIS showed a better correlation with the FC levels, as compared to MES (Meng’s z = − 2.457, *p* = 0.01).

**Table 2:** Inter-rater agreement of the endoscopic indices in the 181 study subjects

Variables	Weighted Kappa^a^	95% CI
Mayo endoscopic subscore	0.78	0.71–0.85
UCEIS	0.62	0.56–0.69
Vascular	0.69	0.57–0.80
Bleeding	0.40	0.31–0.49
Erosions and ulcers	0.74	0.67–0.81

^a^Weighted kappa (all levels disagreement between raters are weighted equally)

*CI* confidence interval, *UCEIS* ulcerative colitis endoscopic index of severity

### Receiver operating characteristics curve analysis

ROC curve analysis indicated a FC cut-off level of 187.0 mg/kg (area under the curve [AUC], 0.883; 95% CI, 0.772–1.000) for predicting complete MH defined as MES value of 0 (Table 3). ROC analysis revealed the equal FC cut-off level of 187.0 mg/kg (AUC, 0.883; 95% CI, 0.772–1.000) for predicting complete MH defined as a UCEIS value of 0 (Table 3). The sensitivity and specificity of the cut-off value of 187.0 mg/kg for complete MH were 0.857 and 0.891, respectively (Table 3).

**Table 3:** Cut-off FC levels for discriminating clinical remission and mucosal healing

	Cut-off FC level (mg/kg)	AUC (95% CI)	*p* value	Sensitivity	Specificity
pMS = 0	1272	0.748 (0.616–0.880)	<0.001	0.857	0.599
pMS = 0–2	2211	0.772 (0.701–0.842)	<0.001	0.875	0.592
MES = 0	187	0.883 (0.772–1.000)	<0.001	0.857	0.891
UCEIS = 0	187	0.883 (0.772–1.000)	<0.001	0.857	0.891

*pMS* partial Mayo score, *MES* Mayo endoscopic subscore, *UCEIS* ulcerative colitis endoscopic index of severity, *FC* fecal calprotectin, *AUC* area under curve, *CI* confidence interval

**Fig. 2:**
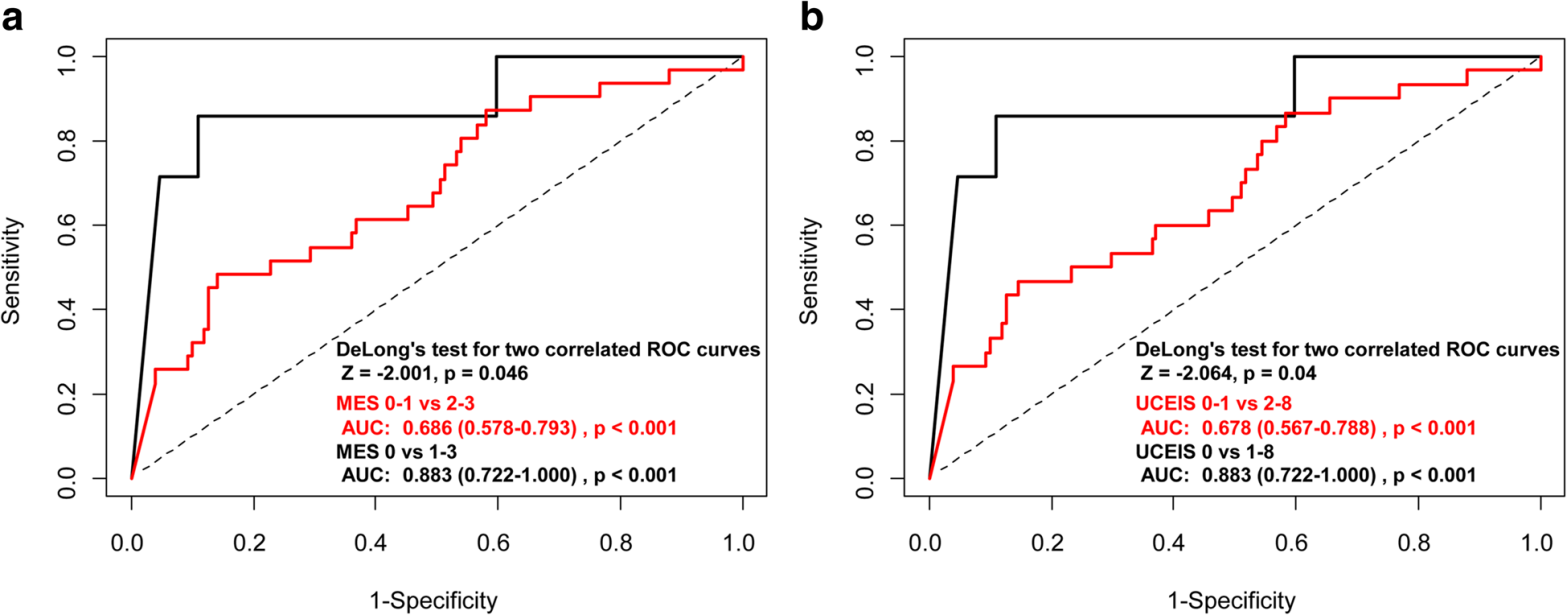
ROC curve analysis showing the AUC of the FC level and comparison between the AUCs (DeLong’s test) of the FC level **a**) for predicting MES = 0 and MES = 0-1; **b**) for predicting UCEIS = 0 and UCEIS = 0-1. *FC* fecal calprotectin, *ROC* receiver operating characteristics, *AUC* area under curve, *MES* Mayo endoscopic subscore, *UCEIS* ulcerative colitis endoscopic index of severity, *CI* confidence interval, *Z* coefficient of DeLong’s test

ROC curve analysis showed much higher FC cut-off levels of 1272.0 mg/kg (AUC, 0.748; 95% CI, 0.616–0.880) for predicting complete clinical remission (pMS = 0) and 2211.0 mg/kg (AUC, 0.772; 95% CI, 0.701–0.842) for predicting near-complete clinical remission (pMS = 0–2) (Table 3). The sensitivity and specificity of both cut-off values of 1272.0 mg/kg (pMS = 0) and 2211.0 mg/kg (pMS = 0–2) were 0.857 and 0.599, and 0.875 and 0.592, respectively (Table 3). The AUC of the FC level for predicting near-complete MH defined as MES = 0–1 was 0.686 (95% CI, 0.578–0.793; Fig. 2a). In contrast, the AUC values of the FC level for predicting UCEIS = 0, 0–1, 0–2, and 0–3 were 0.883 (95% CI, 0.772–1.000), 0.678 (95% CI, 0.567–0.788), 0.725 (95% CI, 0.640–0.809), and 0.730 (95% CI, 0.653–0.806), respectively. When comparing the AUC of FC levels for predicting MES = 0 and MES = 0-1, the DeLong’s test showed significant difference (Z = −2.001, *p* = 0.046) (Fig. 2a). Similarly, when comparing the AUC of FC levels for predicting UCEIS = 0 and UCEIS = 0-1, the DeLong’s test showed significant difference (Z = −2.064, *p* = 0.04) (Fig. 2b).

**Fig. 3:**
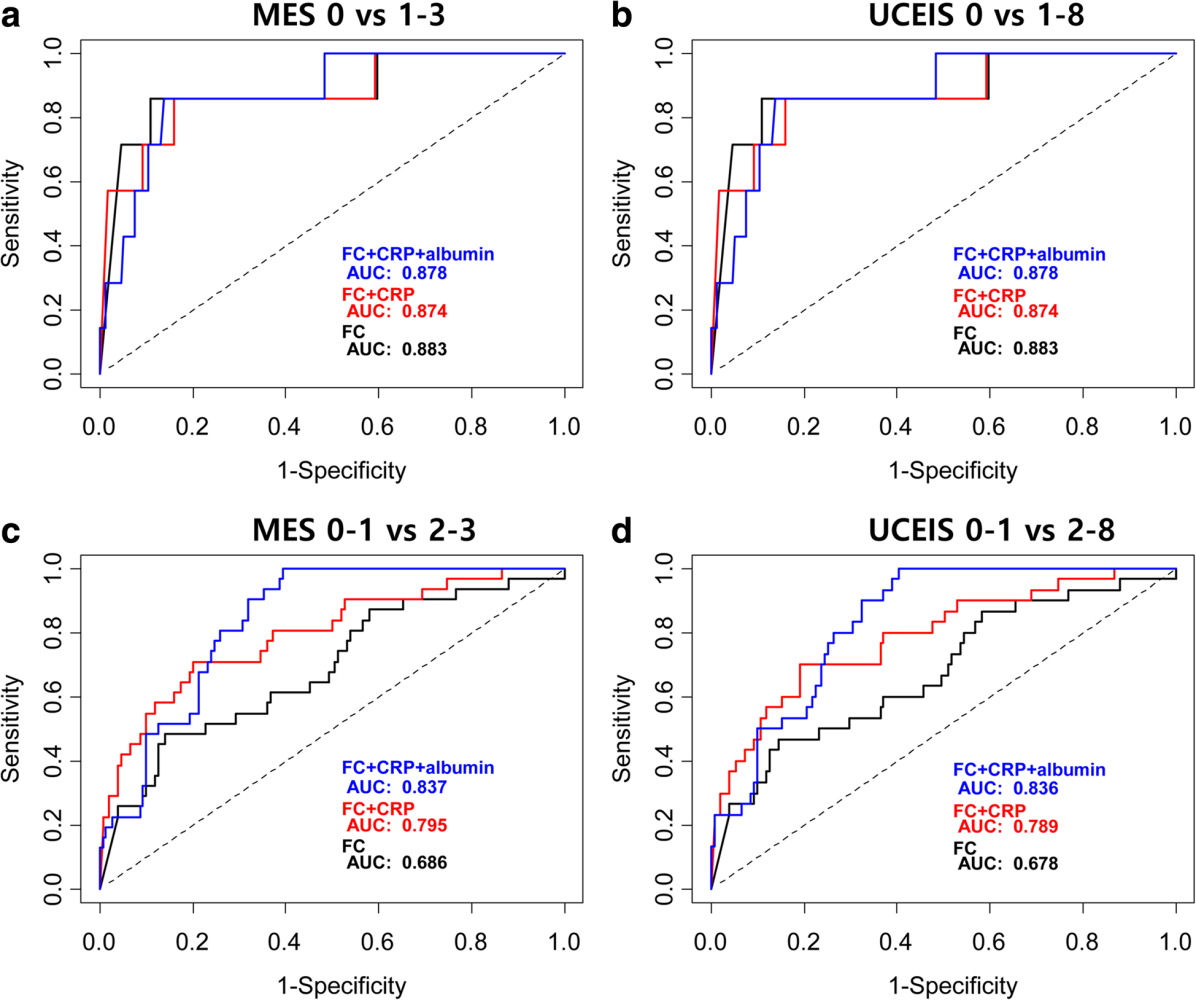
ROC curve analysis showing the AUC of the FC level in combination of biochemical variables for predicting **a**) MES = 0; **b**) UCEIS = 0; **c**) MES = 0-1; **d**) UCEIS = 0–1. *FC* fecal calprotectin, *ROC* receiver operating characteristics, *AUC* area under curve, *MES* Mayo endoscopic subscore, *UCEIS* ulcerative colitis endoscopic index of severity

After combining additional biochemical variables to FC, the AUC of FC + CRP for predicting MES = 0 and MES = 0–1 were 0.874 and 0.795, respectively, and the AUC of FC + CRP + serum albumin for predicting MES = 0 and MES = 0–1 were 0.878 and 0.837, respectively (Fig. 3). A similar trend was observed for UCEIS. The AUC of FC + CRP for predicting UCEIS = 0 and UCEIS = 0-1 were 0.874 and 0.789, respectively, whereas the AUC of FC + CRP + serum albumin for predicting UCEIS = 0 and UCEIS = 0-1 were 0.878 and 0.836, respectively (Fig. 3).

## Discussion

In the present study, we evaluated the correlation of the FC levels with the 2 endoscopic severity indices commonly used in patients with UC. To our knowledge, this is the first study to directly compare between the correlation coefficients of MES and UCEIS with FC levels.

Our study showed a significant correlation of the FC level with both the CRP and serum albumin levels. Because both FC and CRP are representative inflammatory biomarkers used in monitoring disease activity in patients with IBD, they were expected to exhibit a good correlation, consistent with previous studies [19, 22]. However, no previous study has indicated a significant correlation between the FC level and the serum albumin level. Although protein loss from the gut and malnutrition may influence the serum albumin level, the albumin level is also known to decrease in active disease and to be negatively correlated with UC disease activity; this could explain the negative correlation between the serum albumin and FC levels [30, 31]. Moreover, the significant correlation between FC and serum albumin in our study might reflect the high proportion of moderate to severe disease activity in our cohort. In addition, when considering the clinical activity of UC, our study showed a significant correlation between FC and pMS, consistent with the results of the study by Theede et al. [14].

With regard to the endoscopic activity indices, both MES and UCEIS exhibited significant correlations with the FC level in our study, consistent with the observations from previous studies [11, 14, 18–20, 22, 23, 32–34]. Only one previous study by Theede et al. evaluated the correlation of FC levels with both MES and UCEIS [14]. However, the researchers did not directly compare the correlation coefficients of the FC levels with MES and UCEIS. To further determine the significance of the difference between the correlation coefficients of the 2 endoscopic indices with the FC level, we assessed Meng’s z score in the present study [28]. Based on our results, FC levels were better correlated with UCEIS than with MES. However, our results should be validated in a larger number of subjects and in different ethnic groups.

Another strength of our study was the use of endoscopic scores graded by 2 certified expert endoscopists. To increase the inter-rater agreement on scoring, initial training with 50 other cases was performed. As a result, both MES and UCEIS showed substantial inter-rater agreement (weighted Kappa [95% CI] of 0.78 [0.71–0.85] and 0.62 [0.56–0.69], respectively), which was comparable to the inter-rater agreement of UCEIS (weighted Kappa [95% CI]: 0.5 [0.49–0.52]) determined by Travis et al. [17, 35]. The ‘bleeding’ item of the UCEIS had the lowest weighted Kappa coefficient, consistent with that reported by Travis et al. [17, 35]. Travis et al., explained that this may be due to the misinterpretation of the descriptions and the confusion of spontaneous bleeding with contact bleeding [17]. Given the fact that the majority of our study patients had moderate to severe disease activities, the difficulties in differentiation between spontaneous bleeding and contact bleeding especially in this retrospective setting might have contributed even more to the low inter-rater agreement of the ‘bleeding’ item. Despite the presence of significant correlations between UCEIS and MES, UCEIS tended to have a weaker inter-rater agreement as compared to MES, which may possibly be due to the larger number of items used for scoring UCEIS. Nevertheless, further studies are warranted for the comparison of MES and UCEIS.

The cut-off FC level for MH defined as MES = 0 and UCEIS = 0 was equal (187.0 mg/kg). This level is similar to the cut-off level of 192 mg/kg for MH defined as MES = 0 and for MH defined as UCEIS = 0 in the study by Theede et al. [14], and 200 mg/kg for MH defined as MES = 0 in the study by Takashima et al. [20]. Therefore, the cut-off FC level for the strict definition of MH appears to be similar across ethnicities. However, in the present study, the cut-off FC level for complete clinical remission defined as pMS = 0 was relatively high (1272.0 mg/kg), as compared to the cut-off value of 192 mg/kg reported in the study by Theede et al. [14]. This discrepancy may be related to the subjectiveness of the pMS, which is estimated based on bowel frequency, presence of rectal bleeding, and the physician’s global assessment; such bias may reduce the reliability of pMS as a marker of actual mucosal inflammation.

Based on the ROC curve analyses, the AUC of FC for predicting complete MH defined as MES = 0 and UCEIS = 0 had the greatest power of predictability and was comparable with the results of Theede et al. [14]. In comparison between AUC levels, FC had a better predictability of complete MH (MES = 0, UCEIS = 0) rather than near-complete MH (MES = 0-1, UCEIS = 0-1) (DeLong’s test Z = −2.001 [*p* = 0.046], Z = −2.064 [*p* = 0.04]). In addition, AUC could be increased when the FC level was combined with other biochemical markers for predicting near-complete MH (MES = 0-1, UCEIS = 0-1), consistent with the study by Lin et al. [19]. However, AUC could not be increased with the combination of biochemical markers for predicting complete MH (MES = 0, UCEIS = 0). Based on our ROC curve analyses, we could conclude that FC significantly predicts complete MH in UC patients. Although other biochemical markers in combination to FC enhanced the predictability of near-complete MH (MES = 0-1 and UCEIS = 0-1), combination of other biochemical markers to FC did not seem to affect the predictability of complete MH. Future studies on different patient population should further explore the predictability of FC and biochemical markers for predicting MH in UC patients.

The present study had certain limitations of note. First, the analysis was retrospective. Yet, our IBD registry was prospectively maintained and the pMS was also prospectively recorded at each patient visit. Second, despite the presence of statistical significance, the correlation coefficients between the FC level and clinical activity index and endoscopic activity indices appeared to be lower than the values reported in other studies. This can be explained by the variable intervals between the time of FC level measurement and clinical and endoscopic index scoring, which is a major limitation of a retrospective study. For instance, patients with shorter intervals between FC level measurement and endoscopy showed better correlation coefficients (for interval ≤ 14 days: *r* = 0.392, *p* < 0.001; for interval > 14 days: *r* = 0.199, *p* = 0.180). The differences in the FC measurement modalities and study subjects might also have contributed to the different results between the present results and those of previous studies. In addition, the difference between the modalities of endoscopy (full colonoscopy and flexible sigmoidoscopy) might have also contributed to this issue. Third, the FC levels in the present study were relatively higher than those in other studies. The median FC level in our study was 1518.0 mg/kg (IQR, 360.0–4205.0 mg/kg), which was slightly higher than the median FC level (1020.0 mg/kg; IQR, 601.5–1617.5 mg/kg) in the study by Ho et al. in an acute severe UC setting [36]. This may be explained by the selection bias due to the higher severity of UC patients referred to our hospital, which is a tertiary referral center with the largest IBD center in Korea. Although there was no recommended method to exactly measure FC levels below 100 mg/kg, nevertheless, we decided to include cases with the FC levels <100 mg/kg (FC <100 mg/kg were set as 100 mg/kg) to minimize additional selection bias. Finally, the correlation between the FC level and histologic inflammation was not evaluated in our study.

## Conclusions

In conclusion, FC levels exhibit significant correlations with the clinical activity index, endoscopic activity indices, and other serum inflammatory biomarkers in Korean patients with UC. FC is highly predictive of complete mucosal healing in UC patients. In particular, UCEIS shows a better correlation with the FC level as compared to the MES. Thus, FC could be used as a reliable noninvasive indicator for evaluating disease activity and mucosal healing in patients with UC.

## Abbreviations

AUC: Area under the curve
CI: Confidence interval
CRP: C-reactive protein
ESR: Erythrocyte sedimentation rate
FC: Fecal calprotectin
IBD: Inflammatory bowel disease
IQR: Interquartile range
MES: Mayo endoscopic subscore
MH: Mucosal healing
NR: Normal range
pMS: Partial Mayo score
ROC: Receiver operating characteristics
UC: Ulcerative colitis
UCEIS: Ulcerative colitis endoscopic index of severity
